# Combined venous and arterial thrombosis revealing underlying myeloproliferative disorder in a young patient: a case report

**DOI:** 10.1186/s13256-020-02593-5

**Published:** 2021-02-16

**Authors:** Rime Benmalek, Hanane Mechal, Hatim Zahidi, Karim Mounaouir, Salim Arous, Mohamed El Ghali Benouna, Abdenasser Drighil, Rachida Habbal

**Affiliations:** Cardiology department, Hospital University Center Ibn Rochd, Casablanca, Morocco

**Keywords:** Myeloproliferative neoplasms, Polycythemia vera, Complications, Acute myocardial infarction, Cerebral venous thrombosis, Young adult

## Abstract

**Background:**

Myeloproliferative neoplasms (MPNs) such as polycythemia Vera (PV) and Essential Thrombocythemia (ET) can be associated with a high risk of both venous and arterial thrombosis. However, the co-existence between these two complications is very rare and has never been described before, especially in young adults with no known history of MPNs.

**Case presentation:**

We report the case of a 39 year-old Caucasian Moroccan male patient without cardiovascular risk factors (CVRF), who presented with acute chest pain. He also suffered from a severe headache since 2 weeks. Electrocardiogram (ECG) showed ST segment elevation myocardial infarction in the posterolateral leads. Cerebral Computed Tomography (CT) scan revealed subarachnoid hemorrhage (SAH), and cerebral Magnetic Resonance Angiography (MRA) found a Superior Sagittal Sinus Thrombosis (SSST). Routine blood tests showed raised hemoglobin and hematocrit in addition to leukocytosis and thrombocythemia. His coronary angiography revealed a thrombus in the ostial left circumflex artery (LCX). Further testing revealed positive Janus kinase 2 (JAK2) V617F mutation and low erythropoietin level, confirming the diagnosis of PV according to the 2008 World Health Organization (WHO) criteria. Antithrombotic and anti-ischemic treatments, in addition to myelosuppressive therapy with hydroxyurea, were initiated with a good clinical and biological evolution.

**Conclusion:**

This case shows that MPNs are an important cause of thrombosis, especially in young patients with no other risk factors. Early diagnosis and appropriate management are fundamental before the occurrence of life-threatening complications that can sometimes present in unusual forms associating arterial and venous thrombotic events.

## Background

Myeloproliferative neoplasms (MPNs) are a group of hematological disorders characterized by abnormal proliferation of all hematopoietic bone marrow elements responsible for a state of hyperviscosity and thrombocytosis. Thromboembolic and cardiovascular events can be life-threatening complications in this disease [1].

Among thrombotic complications, arterial events such as acute myocardial infarction (AMI) and stroke occur in more than two-thirds of cases, often in the setting of atherosclerosis and traditional cardiovascular risk factors (CVRF) [2]. Rarely, arterial thrombotic events affect young adults with no CVRF, and serve as a starting point for revealing one of the less common causes of arterial thrombosis in young patients : MPNs. Moreover, MPNs are also responsible for venous thrombotic complications in one-third of cases, including cerebral venous thrombosis (CVT) [2].

Nevertheless, the association between these two thrombotic complications is very rare and has never been described before.

This case outlines the importance of pointing out MPNs as an important cause of thrombosis in young patients without CVRF. It emphasizes the necessity of early recognition of the disease, which can sometimes be initially present in unusual forms associating with arterial and venous events to initiate proper management strategies.

## Case presentation

We report the case of a 39 year-old Caucasian Moroccan male patient with no traditional cardiovascular risk factors nor for venous thromboembolism, who presented to the Emergency department with acute retrosternal constrictive chest pain radiating to the left shoulder, that started 14 h ago, associated with stage II dyspnea according to New York Heart Association (NYHA) classification and with a severe headache that he described as recurrent since 2 weeks, with no associated neurologic deficit nor meningeal syndrome.

**Fig. 1:**
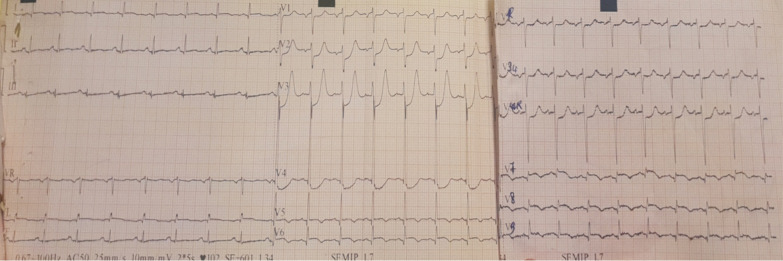
18-leads Electrocardiogram (ECG) At admission showing ST segment-elevation with a necrosis Q-wave in posterolateral leads with mirror view in anteroseptoapical leads.

**Fig. 2:**
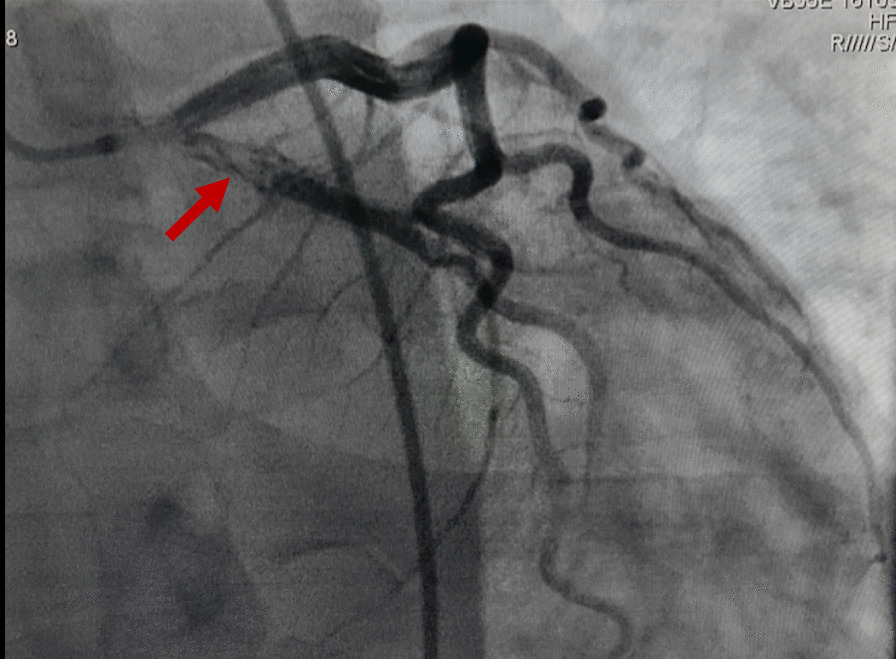
Coronary angiogram in right anterior oblique (RAO) caudal projection showing a thrombus in the ostial Left circumflex artery (LCX) (Red arrow) in addition to a short and spastic Left main coronary artery (LM).

Physical examination found a conscious patient with facial erythrosis, a blood pressure of 140/75 mmHg, a pulse of 103 beats/min (regular), and saturation on room air of 98% with normal heart, neurological, chest and abdominal examination. The Electrocardiogram (ECG) on admission (Fig. 1) showed ST segment-elevation with a necrosis Q-wave in posterolateral leads (V5–V6, DI-aVL, V7–9) and ST depression in V1–4 leads (mirror view). Transthoracic Echocardiography (TEE) revealed hypokinesia of lateral and inferior walls, with a Left Ventricular Ejection Fraction (LVEF) of 40% and increased filling pressure, in addition to a severe ischemic mitral regurgitation and a moderate tricuspid insufficiency with an estimated transvalvular gradient of 77 mmHg, Right ventricular function was normal. The coronary angiography (Fig.2) revealed a thrombus in the ostial Left circumflex artery (LCX) in addition to a short and spastic left main coronary artery (LM), the rest of the arteries were normal with no sign of atherosclerosis.

Given the severe headache not responding to analgesics, a cerebral nonenhanced CT scan was performed, showing blood in the occipital horns of both lateral ventricles with no diffuse SAH (Fisher grade 4).

**Fig. 3:**
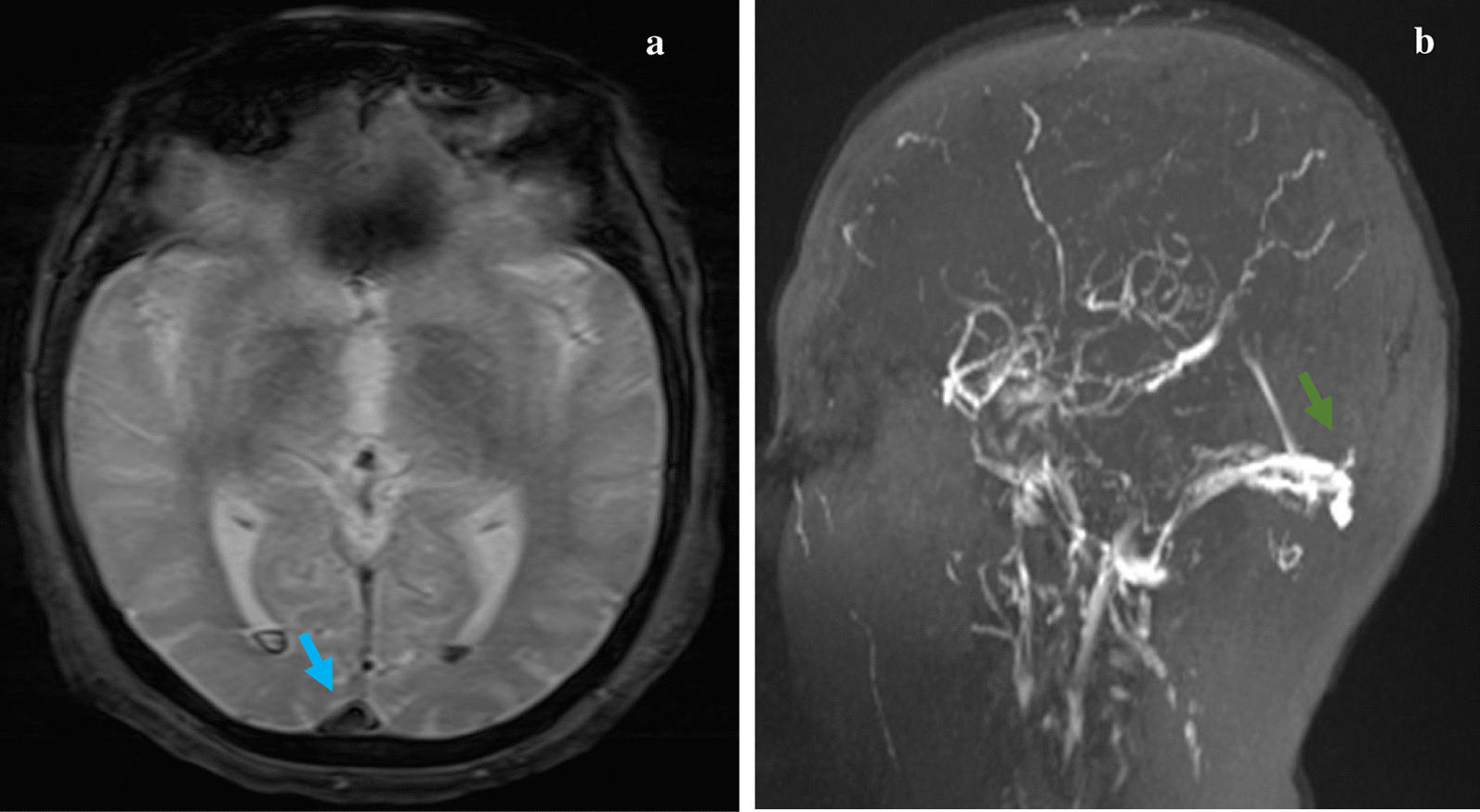
**a** Axial T2*GE magnetic resonance imaging showing a low-attenuating thrombus within the superior sagittal sinus with “empty-delta” sign (blue arrow) and hypointense areas in occipital horns of both lateral ventricles corresponding to subarachnoid hemorrhage. **b** Sagittal magnetic resonance venography showing the filling defect in the superior sagittal sinus indicative of Superior Sagittal Sinus Thrombosis (SSST) (green arrow)

It was then followed by a Cerebral Magnetic Resonance Angiography (MRA) where a Superior Sagittal Sinus Thrombosis (SSST) was discovered in addition to the intraventricular hemorrhage (Fig. 3).

Baseline Laboratory investigations of the patient are shown in Table 1.

**Table 1:** Laboratory investigation of the patient on admission

Parameter	Result	Normal range
Troponin I	82 ng/ml	< 0.1 ng/ml
Aspartate aminotransferase (AST)	27 U/L	5–30 U/L
Alanine aminotransferase (ALT)	62 U/L	5–30 U/L
Lactate dehydrogenase (LDH)	276 U/L	50–150 U/L
Hemoglobin (Hb)	20.2 g/L	13–16 g/L
Hematocrit (Ht)	0.64	0.41–0.50
Red blood cells (RBC)	7.3 T/L	4.7–6.1 T/L
White blood cells (WBC)	22.53 G/L	4–10 G/L
Neutrophils	18.71 G/L	2–8 G/L
Platelets	409 G/L	150–400 G/L
Prothrombin time (PT)	89%	70–100%
Fibrinogen	2.3 g/L	1.8–4 g/L
Urea	1.7 mmol/L	1.2–3 mmol/L
Creatinin	1.0 mg/dL	0.7–1.3 mg/dL
Na+	137 mmol/L	135–145 mmol/L
K+	4.1 mmol/L	3.5–5 mmol/L
Ca++	2.2 mmol/L	2–2.6 mmol/L

The bone marrow examination showed an increase in all cell lineages, suggestive of a myeloproliferative disorder. Further testing revealed positive JAK2/V617F mutation and low erythropoietin level (1.2 mU/ml), cytogenetic study for BCR/ABL1 rearrangement was negative. The tests for Antinuclear Antibodies (AAN), antiphospholipid antibodies (APA), anticardiolipin antibody, and lupus anticoagulant were negative. Findings in abdominal ultrasound were unremarkable with no hepatosplenomegaly. Based on these results, the diagnosis of PV was confirmed accordingly to the World Health Organization (WHO) revised criteria (Table 2) [3].

**Table 2:** World Health Organization revised criteria for the diagnosis of Polycytemia Vera [3]

Major criteria
1. Hb >18.5 g/dL (men)/>16.5 g/dL (women) or Hb or Hct >99th percentile of reference range for age, sex, or altitude of residence or RBC mass >25% above mean normal predicted or Hb >17 g/dL (men)/>15 g/dL (women) if associated with a sustained increase of ≥2 g/dL from baseline that cannot be attributed to correction of iron deficiency
2. Presence of *JAK2* V617F or *JAK2* exon 12 mutation
Minor criteria
1. Trilineage proliferation in Bone Marrow
2. Subnormal Erythropoietin level
3. Endogenous erythroid colony growth
PV diagnosis =2 major criteria and 1 minor criterion **OR** the first major criterion and 2 minor criteria

*Hct* hematocrit, *Hb* hemoglobin, *PV* polycythemia vera, *RBC* red blood cell

For his myocardial infarction, the patient was first treated with ramipril, bisoprolol, atorvastatin and furosemide in addition to low dose aspirin in concertation with the neurologists, after a repeat cerebral CT scan that showed stationary subarachnoid hemorrhage (SAH) after 48 hours. Cytoreductive therapy was also initiated, starting with 15 mg/kg per day of hydroxyurea.

Given the patient’s high thrombotic risk, and his concomitant cerebral venous thrombosis (CVT), we cautiously started anticoagulation (AC) therapy. We initiated, in close collaboration with the neurology team, low doses of intravenous unfractionated heparin, starting with 18 UI/Kg/D with a target partial thromboplastin time (PTT) of 1.5 times the control value.

On the second week, oral AC with Vitamin K Antagonists (VKA) was started, after no evidence of worsening of SAH, with a lower International Normalized Ratio (INR) target to 2.0–2.5.

The patient’s progress was favorable after 3 weeks of treatment and a close neurogical monitoring, with clinical improvement, and progressive correction of hematological parameters. After 21 days, the repeat CT scan showed an important decrease of the SAH secondary to the CVT.

He was discharged from hospital, with dual therapy associating acenocoumarol and low dose aspirin. He was then followed-up as outpatient by a multidisciplinary team of cardiologists, neurologists and hematologists, with a good clinical evolution and a complete regression of his CVT and SAH.

## Discussion

MPNs are a group of hematological disorders, including PV, myelofibrosis (MF) and Essential Thrombocytemia (ET), characterized by clonal stem-cell proliferation of all three lineages [2]. Polycythemia Vera (PV) is a rare disease with an incidence of 2.3 per 100,000 persons per year, with a higher incidence among men of more than 60 years [4], exceptional in younger individuals under 40 years, like our patient. Approximately 96% of patients with PV have a mutation of the JAK2 gene [2]. The diagnosis of PV is based on the WHO’s revised criteria (Table 2) [3].

The disease can be an incidental finding after a routine blood test or present with symptoms mainly related to thrombosis and/or hemorrhage. Indeed, PV is characterized by increased RBC mass resulting in blood hyperviscosity and a higher risk of thrombosis. Pathogenesis of the thrombophilic state acquired in PV is complex and multifactorial and can be summed up in two main mechanisms: The first one is the expression of a prothrombotic phenotype by the abnormal MPN clone–derived blood cells, and the other involves the inflammatory response of host vascular cells that become procoagulant [1]. Moreover, disease related factors, such as increased blood cell counts, and mostly the presence of the JAK2 mutation, can interact with patient related factors, such as age, history of previous thrombotic events, obesity, hypertension, hyperlipidemia, thrombophilia and other known risk factors for thromboembolism increasing the risk of thrombosis.

In our case, the patient had no history of traditional cardiovascular nor venous thromboembolism risk factor.

Thrombotic complications can be classified into 2 categories : microvascular and macrovascular. Microvascular complications (e.g : Erythromelalgia, headache, visual disturbance, tinnitus…) are the result of thrombus formation in small blood vessels, whereas macrovascular complications are the consequence of thrombi formation in big arteries or veins, and are more serious, accounting for 45% of deaths in PV patients [5]. Events involving the arterial system represent two third of the events and include ischemic stroke, Acute Myocardial Infarction (AMI), and peripheral arterial thrombosis. Venous thrombotic events (VTE) are not as frequent (one third), and are represented by deep vein thrombosis, pulmonary embolism, intraabdominal, and CVT [6].

These thrombotic events, particularly arterial, can occur before diagnosis and reveal the disease in almost one fifth of the cases, and can be the leading cause of mortality among patients with PV [7]. A retrospective international study of 1545 patients found thrombotic complications responsible for death in 9% of patients [7], and another study of 1213 patients reported that 30% of 145 arterial thrombotic events and 11% of 87 VTE were fatal, causing 24% and 6% of all deaths [8].

Our case reports the unique situation of a patient with no family history and no cardiovascular risk factors who experienced both major arterial and venous thrombosis, that eventually revealed PV after a thorough evaluation.

Despite the association of PV with coronary artery disease, its presentation as AMI remains very rare, and the association with CVT is exceptional. In the literature, the majority of cases were young males with minimal coronary occlusion, like it was the case for our patient. Rossi et al. studied 149 patients with PV for 10 years and found that 11.4% developed MI [9]. Benita et al reported the case of a 30-year-old male man who died from AMI as initial manifestation of PV. On autopsy, no atherosclerotic changes nor thrombotic occlusion were found on the coronary arteries, only a marked intimal proliferation leading to multiple occlusions [10].

On the other hand, CVT represent an even rarer complication of PV that can lead to death in up to 8.3% of patients as reported by Ferro et al [11]. In PV patients, it is often the consequence of raised venous and capillary pressure due to the hyperviscosity state that results in a lower cerebral perfusion, responsible of ischemic injury and cytotoxic edema. Moreover, Thrombosis of cerebral sinuses causes intracranial pressure that can cause parenchymal hemorrhage and vasogenic and cytotoxic edema [12]. Our patient had in addition to his MI, a SSST CVT revealed in the MRA associated to a meningeal hemorrhage. The co-existence between both arterial and venous thrombosis in the same patient was never described before.

Several mechanisms of such major thrombotic events in patients without risk factors are described. Among them, an increased hematocrit> 45% is associated to an increased risk of cardiovascular events [13], which can be explained in our patient who had an Ht rate of 64%. Similarly, quantitative and qualitative platelet abnormalities as well as leukocytosis, are at a higher risk of AMI [14], like our patient who had in addition to polycythemia, an important leukocytosis and a slight thrombocythemia. Recently, the JAK2 V617F was found to be an independent risk factor for major vascular events [15], which was also the case in our patient. Finally, other risk factors such as older age, prior history of thrombosis, and cardiovascular risk factors, have been assessed in multiple studies [8].

Generally the treatment of MI secondary to MPNs disorders is a challenge since it requires careful attention to maintain the balance between the hemorrhagic and thrombotic risk. A multidisciplinary management between the cardiologist, interventional cardiologist and the hematologist should be undertook. Percutaneous reperfusion associated with antithrombotic treatment followed by cytoreductive therapy is recommended. Hydroxyurea is suggested as first-line myelosuppressive treatment, and anagrelide is recommended as second-line therapy [16]. As for our patient, coronary angiography showed no critical stenosis requiring reperfusion, however, it revealed a thrombotic lesion of the LCX. Antiplatelet therapy consisting of low dose Aspirin was initiated after stationary SAH in the repeat cerebral CT scan performed at day 2. Considering the patient’s concomitant CVT and his high thrombotic risk, we carefully started AC despite his SAH, in concertation with the neurologists, and after performing a repeat CT brain scan that showed regression of the SAH. In a limited number of studies, AC was found to be beneficial for cases of CVT complicated by SAH. In Rodallec et al review, the authors suggested that AC was safe, even in patients with cerebral hemorrhage [17]. On the 5th day, we initiated low doses of intravenous unfractionated heparin, starting with 18 UI/Kg/D with a target PTT of 1.5 times the control value. On the second week, oral AC with acenocoumarol was started, after no evidence of worsening of SAH, with a lower International Normalized Ratio (INR) target to 2.0–2.5.

The proper time to start AC in such cases was not clearly defined in the literature, and a delay of 4–33 days was observed after the onset of symptoms [18].

In our case, knowing that SAH was secondary to CVT, we started AC early and achieved a good recovery in the patient. After 3 weeks the repeat CT scan showed an important decrease of the SAH. The patient was discharged under dual therapy (acenocoumarol+Aspegic) associated with hydroxyurea, and kept being followed-up as outpatient by a multidisciplinary team with a good clinical and neurological progress.

As for the oral anticoagulation, recent studies showed that direct oral anticoagulants (DOACs) such as rivaroxaban and apixaban, can be a good alternative to VKA for the treatment of MPNs-associated VTE. In his review, De Stefano demonstrates the efficacy and safety of DOACs, with less thrombosis recurrence, suggesting that patients with VTE at higher bleeding risk should be considered for treatment with DOACs instead of VKA [19]. These findings were confirmed by a recent cohort study that showed that in MPN patients with VTE, the use of DOACs was associated with very low recurrence rates of VTE and no major bleeding complication [20]. In our patient, VKA were preferred over DOACS for cost and convenience reasons.

Regarding the prognosis, in a retrospective multicenter study of 497 patients, De Stefano et al. studied the recurrence rate of thrombotic events in patients with MPNs who had previously suffered arterial or venous thrombosis or both. They reported that treatment associating antiplatelet and VKA showed effectiveness in preventing recurrence and re-thrombosis of 58% and 68% respectively. Their study also showed that the best preventive strategy in these cases is represented by the adjunction of cytoreductive therapy [21]. Another Korean study showed that previous thrombotic history and a positive JAK2 V617F mutation, like in our patient’s case, were associated to an increased 10-year cumulative rate of thrombohemorrhagic events in patients with MPNs [22].

## Conclusion

Major arterial and/or venous thrombotic events can present as the first manifestation of a more general disease. When such complications occur in a young patient with no risk factors, alternative etiologies should be investigated, among them, MPNs. By describing a unique case of a young patient who presented both AMI and CVT that eventually revealed PV, this report highlights the importance of careful assessment of blood cell count abnormalities in such patients to enable early diagnosis and appropriate multidisciplinary management between cardiologists, neurologists and hematologists, that is essential to reduce the risk of further thrombotic events and prevent morbidity and mortality.

## Data Availability

The published information is available from the corresponding author on reasonable request.

## Abbreviations

MPNs: Myeloproliferative neoplasms
PV: Polycythemia Vera
ET: Essential Thrombocytemia
CRVF: Cardiovascular risk factors.
WHO: World Health Organization
ECG: Electrocardiogram
TEE: Transthoracic Echocardiography
CT: Computed Tomography
MRA: Magnetic Resonance Angiography
SSST: Superior Sagittal Sinus Thrombosis
SAH: Subarachnoid hemorrhage
LCX: Left circumflex artery
LM: Left main coronary artery
JAK2: Janus kinase 2
AMI: Acute myocardial infarction
CVT: Cerebral venous thrombosis
VTE: Venous thrombotic events
NYHA: New York Heart Association
RBC: Red blood cells
WBC: White blood cells
AC: Anticoagulation
VKA: Vitamin K antagonists
